# Navigating the Crisis of White Cord Syndrome in a Diabetic Female Patient After Cervical Spine Surgery: A Case Report

**DOI:** 10.7759/cureus.100049

**Published:** 2025-12-25

**Authors:** Harold L Rivera-Alvarado, Guillermo Garcia-Vargas, Julián J Zayas-Vélez, Amanda Cintron-Santiago, Myrna Morales-Franqui, Emil A Pastrana, Hector Torres, Maria J Crespo

**Affiliations:** 1 Anesthesiology, University of Puerto Rico-School of Medicine, San Juan, PRI; 2 Neurosurgery, University of Puerto Rico-School of Medicine, San Juan, PRI; 3 Physiology, Anesthesiology, University of Puerto Rico-School of Medicine, San Juan, PRI

**Keywords:** cervical spinal surgery, diabetes, ischemic-reperfusion injury, neuroanesthesia, white cord syndrome

## Abstract

We report the case of a 72-year-old woman with a medical history of asthma, hypothyroidism, and type 2 diabetes mellitus who underwent C4-C6 corpectomy with fibular osseous graft replacement and C2-T1 decompressive laminectomy with C2-T1 lateral fusion. The procedure also included posterior spinal fixation from C2 to T1 using transpedicular screws, along with decompressive laminectomies at the C2-T1 levels for symptomatic multilevel cervical stenosis. Intraoperatively, the patient developed a sudden loss of all neuromonitoring signals, consistent with an acute neurological insult. High-dose corticosteroids were administered to reduce inflammation and edema, and blood pressure was aggressively managed with vasopressors to maintain adequate mean arterial pressure. Despite these interventions, the patient demonstrated postoperative neurological deterioration. The patients’ motor strength improved in upper and lower extremities to 2/5 and 1/5, respectively, during evaluation in the postoperative care unit; however, neurological recovery plateaued during her subsequent stay in the neuro-intensive care unit, prompting transfer to an inpatient rehabilitation facility. In this case report, we present the intraoperative and postoperative management of an elderly diabetic patient who developed acute neurological deficits consistent with white cord syndrome (WCS) following cervical spine surgery, with particular emphasis on the interplay between perioperative hyperglycemia, high-dose corticosteroids, and WCS pathophysiology.

## Introduction

White cord syndrome (WCS) is a rare complication following spinal cord decompression surgery, with very few cases identified [1]. It is characterized by the unexpected postoperative worsening of neurological status despite successful decompression and the appearance of a hyperintense signal on T2-weighted magnetic resonance imaging (T2W-MRI), a feature that defines and gives the syndrome its name [2,3]. The timing of symptom onset and associated imaging findings is variable, with presentations ranging from immediately after surgical decompression to several days or even weeks postoperatively [4].

Although the exact pathophysiological basis for WCS remains unclear, several mechanisms have been proposed. Accumulating evidence suggests that ischemia-reperfusion injury is the leading mechanism involved in the etiology of this syndrome [2,3]. This process is characterized by the production of free radicals and reactive oxygen species (ROS) during the restoration of blood flow, which paradoxically results in tissue damage. An exaggerated inflammatory cascade involving pro-inflammatory cytokines and leukocyte infiltration may contribute to spinal cord inflammation, edema, and neuronal injury [5]. Postoperative edema can compress the spinal cord despite adequate surgical decompression. In addition, endothelial disruption during decompression may impair blood flow regulation and increase microvascular permeability, further worsening edema and neurologic deterioration [2].

Although no formal diagnostic criteria for WCS have been established by any medical organization, the diagnosis is primarily clinical, supported by characteristic imaging findings and the exclusion of other potential causes [1,4,6]. A recent review by Epstein et al. [3], however, proposed WCS as a “diagnosis of inclusion” requiring MRI confirmation. WCS typically presents as new or worsening neurological deficits occurring immediately or shortly after spinal decompression surgery, without evidence of direct mechanical trauma, hematoma, microthrombi, or other identifiable complications [1].

While knowledge of the underlying mechanisms of WCS is still emerging, current treatment options remain relatively limited. Initial management typically includes the early administration of high-dose corticosteroids to reduce spinal cord inflammation and edema [1]. Initiating treatment within the first 24 hours may help limit secondary injury. Maintaining a mean arterial pressure (MAP) of at least 85-90 mmHg is also critical to ensure adequate spinal cord perfusion and prevent further ischemic damage [1,7]. The MAP target of 85-90 mmHg was selected based on extrapolation from acute traumatic spinal cord injury (SCI) guidelines, as direct evidence supporting this target in WCS remains limited. This is achieved via vasopressor support and appropriate fluid management. Continuous monitoring of neurological status and therapeutic response is essential, as outcomes vary amongst cases. Some patients recover fully during hospitalization, while others may require transfer to an inpatient rehabilitation facility for continued physical and occupational therapy [8]. Prognosis remains uncertain and varies widely between patients, presenting a significant challenge for clinicians [9].

Although WCS is estimated to occur in less than 1% of all spinal surgeries [10], the true incidence of this condition may be underestimated due to the absence of standardized diagnostic criteria and the uncommon nature of its clinical presentation. As a result, epidemiological data on WCS remain limited. Current management strategies are largely informed by small studies and individual case reports. In this case report, we present the intraoperative and postoperative management of an elderly diabetic patient who developed acute neurological deficits consistent with WCS following cervical spine surgery. This case highlights the risk of unexpected complications during spinal cord decompression procedures, particularly in diabetic patients, for whom elevated oxidative stress is a well-recognized hallmark and may play a key role in the development of WCS.

## Case presentation

**Figure 1:**
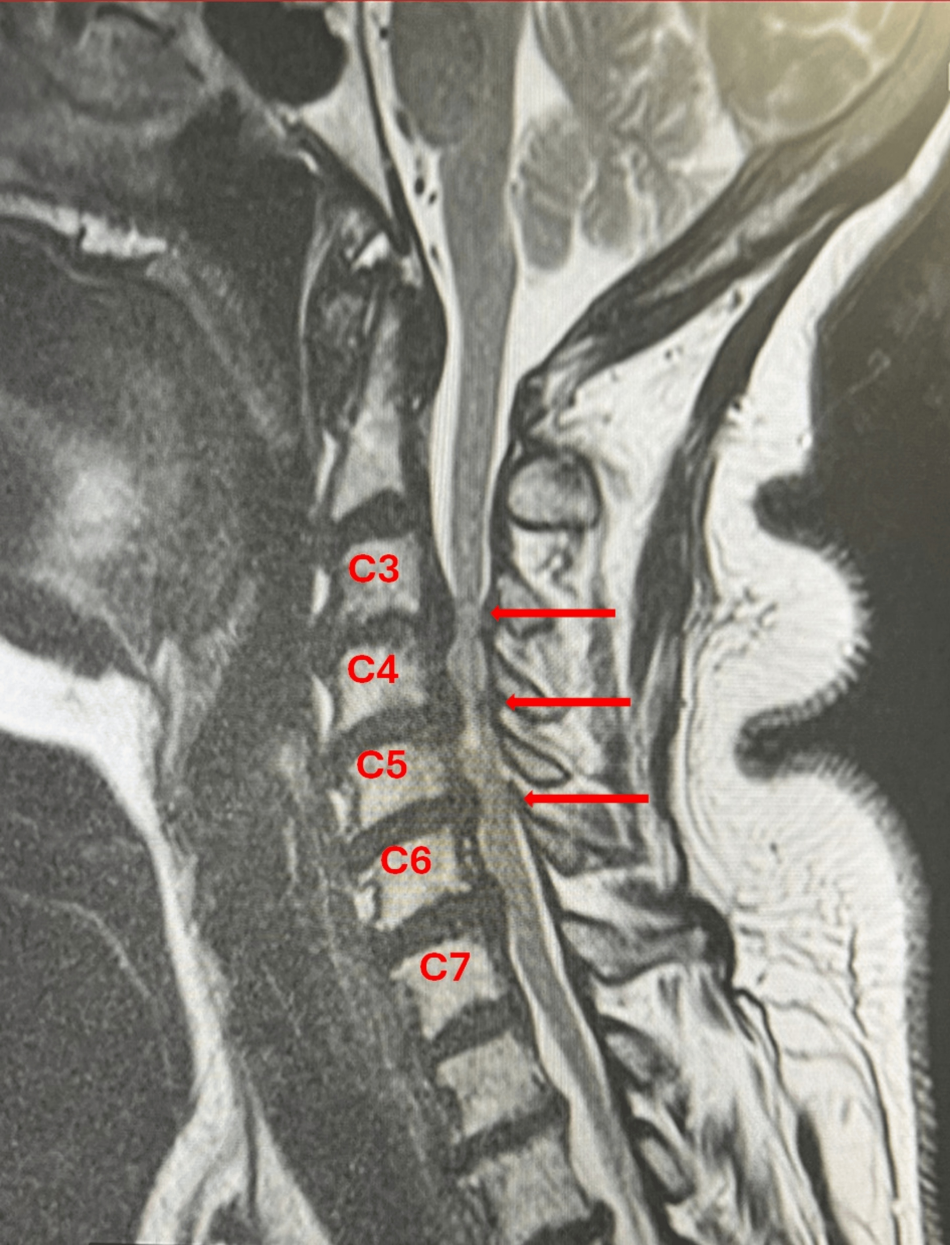
Preoperative cervical spine sagittal T2-weighted MRI. Red arrows show multilevel cervical stenosis.

A 72-year-old woman with a diagnosis of cervical stenosis complicated by myelopathy and radiculopathy was scheduled by the neurosurgery service to undergo an elective C3-C7 anterior cervical discectomy and fusion. Due to neurosurgical team assessment, she underwent a C4-C6 corpectomy with fibular osseous graft replacement and C2-T1 decompressive laminectomy with C2-T1 lateral fusion. Preoperative MRI showed severe cervical stenosis (Figure 1). Her medical history was significant for bronchial asthma and type 2 diabetes mellitus managed with oral agents. Surgical history included bilateral partial salpingectomy, tonsillectomy, and dilation and curettage. At the time of evaluation, home medications included metformin, gabapentin, naproxen, and pravastatin. She reported no known drug allergies and denied tobacco use, alcohol consumption, or recreational drug use.

On preoperative assessment, the patient was alert and oriented to person, place, and time. An American Society of Anesthesiologists (ASA) physical status score of 2 was determined for the patient. Neurological examination showed bilateral upper and lower extremity motor strength of 4/5, numbness of the upper extremities, and 3+ reflexes throughout, with a positive Hoffman sign seen bilaterally. A 20-gauge peripheral intravenous catheter was placed in the right upper extremity and confirmed to be patent. She had fasted for more than eight hours before the procedure. Her height and weight were recorded as 62 inches and 59 kg, respectively.

Preoperative laboratory values are included in Table 1. The preoperative glucose level of 157 mg/dL supports the notion of suboptimal glycemic control, although prior glucose values or longitudinal glycemic data were not available for comparison. Vital signs prior to induction included a blood pressure of 141/93 mmHg, heart rate of 71 beats per minute, and oxygen saturation of 99% on room air. Preoperative medications administered at the request of the surgical team included intravenous (IV) famotidine (20 mg), cefazolin (2 g), and vancomycin (1 g).

**Table 1:** Preoperative laboratory results. SGPT: Serum glutamic-pyruvic transaminase, SGOT: Serum glutamic-oxaloacetic transaminase, AST: Aspartate transaminase, ALT: Alanine transaminase

Test	Result	Normal Range
White Blood Count (X 10^3/uL)	4.1	3.98-10.04
Red Blood Count (X 10^6/uL)	4.3	3.93-5.22
Hemoglobin (gm/dL)	13.2	11.2-15.1
Hematocrit (%)	38	36-44
Platelets (X 10^3/uL)	218	163.0-369.0
Prothrombin Time (seconds)	11.5	9.00-11.50
Partial Thromboplastin Time (seconds)	30.7	22.2-34.0
International Normalized Ratio (INR)	1.1	0.9-1.1
Sodium (mEq/L)	140	135.-145.00
Potassium (mEq/L)	4	3.3-5.1
Chloride (mEq/L)	104	98.0-107.0
Carbon Dioxide, Total (mEq/L)	28	25.0-30.0
Blood Urea Nitrogen (mg/dL)	13	7.0-20.0
Creatinine (mg/dL)	0.8	0.5-1.5
Glucose (mg/dL)	157	70.0-99.0
Alkaline Phosphatase (u/L)	83	38.0-126.0
SGPT (ALT) (u/L)	21	0.0-35.0
SGOT (AST) (u/L)	23	15.0-46.0
Albumin (g/dL)	4.5	3.5-5.0

The patient arrived at the operating room in stable condition and was premedicated with glycopyrrolate (0.2 mg, IV), dexamethasone (4 mg, IV), and midazolam (2.5 mg, IV). ASA monitors were connected, and pre-oxygenation with 100% O2 was administered for three minutes. Induction was achieved with propofol (120 mg, IV), fentanyl (100 µg, IV), lidocaine (100 mg, IV), and succinylcholine (100 mg, IV). Intubation was performed using a McGrath video laryngoscope (Macintosh blade size 3) with manual in-line stabilization. A 6.5-mm endotracheal tube was secured at 21 cm at the right labial commissure. Positive bilateral breath sounds were confirmed via auscultation, and the patient was placed on volume control ventilation with a tidal volume of 400 mL, a respiratory rate of 14 bpm, and positive end-expiratory pressure (PEEP) of 5 mmHg. General anesthesia was maintained with inhaled sevoflurane (1%), lidocaine (2 mg/min, IV), and propofol (100 µg/kg/min, IV). Vital signs remained stable, with MAP initially ranging between 73 and 96 mmHg, temperature of 36°C, pulse rate of 71-95 bpm, and oxygen saturation of 98-100%. Neuromonitoring was established and confirmed functional prior to incision.

**Figure 2:**
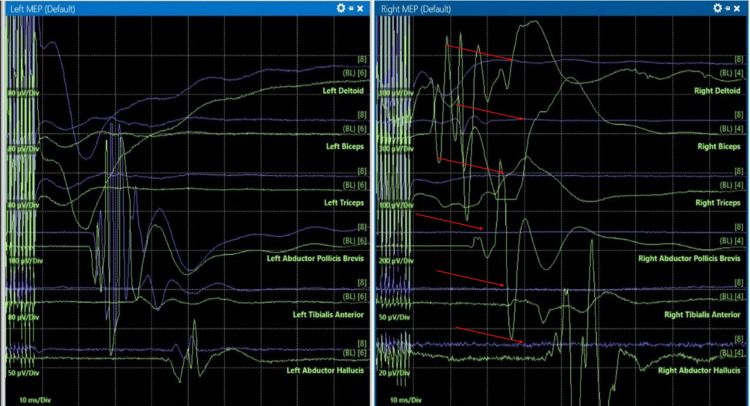
Intraoperative neurophysiological monitoring signals showing a reduction of right-sided motor-evoked potentials during C3-C4 instrumentation. Green: baseline MEPs; purple: live MEPs; red arrows: reduction in responses

**Figure 3:**
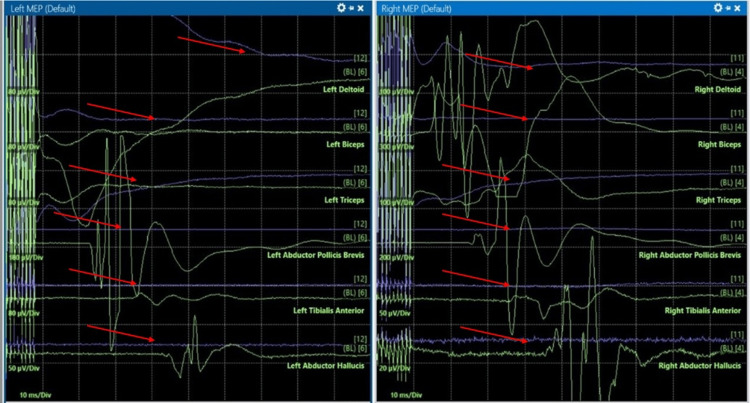
Intraoperative neurophysiological Monitoring Signals showing loss of bilateral motor-evoked potentials during C4-C5 instrumentation. Green: baseline MEPs; purple: live MEPs; red arrows: reduction in responses

During C3-C4 instrumentation (25 minutes after surgical procedure initiation), the patient experienced a reduction of motor evoked potentials (MEPs) in the right upper and lower extremities (Figure 2), while the left side remained intact. Subsequently, during C4-C5 instrumentation (approximately five minutes after C3-C4 instrumentation), bilateral upper extremities somatosensory evoked potentials (SSEPs) were diminished (Figure 3), and complete loss of SSEPs and MEPs occurred following C4 corpectomy (approximately 55 minutes after procedure initiation). In response to the neuromonitoring alert, surgical manipulation was immediately stopped. Reversible technical and anesthetic causes were systematically evaluated in coordination with the surgical and neuromonitoring teams. Given the persistent signal loss, the National Acute Spinal Cord Injury Study (NASCIS) protocol was initiated. A bolus of 30 mg/kg IV methylprednisolone was administered over a 15-minute period, followed by a maintenance infusion of 5.4 mg/kg/hr for 23 hours. In addition, sevoflurane was discontinued (MAC 0.0), MAP was maintained above 90 mmHg using vasopressor support, and ventilation was adjusted to maintain normocarbia.

Blood glucose levels were monitored every hour, and midazolam (total of 5 mg in four doses of 1.25 mg) was given as needed to reduce the risk of awareness. Following the completion of the anterior segments of the procedure, which took approximately three hours, a radial artery line was placed to carefully monitor the patient’s hemodynamics. To maintain adequate MAP, norepinephrine (3 µg/min, IV) was administered. The patient was then repositioned on the Allen table to begin the posterior cervical aspect of the procedure, which lasted approximately 2.5 hours. Intraoperative glucose levels ranged from 222 to 310 mg/dL. Two doses of subcutaneous insulin (10 units four hours after the start of the procedure and 15 units at the end) were administered to maintain appropriate glucose levels. The total surgery time was five hours and 30 minutes.

**Figure 4:**
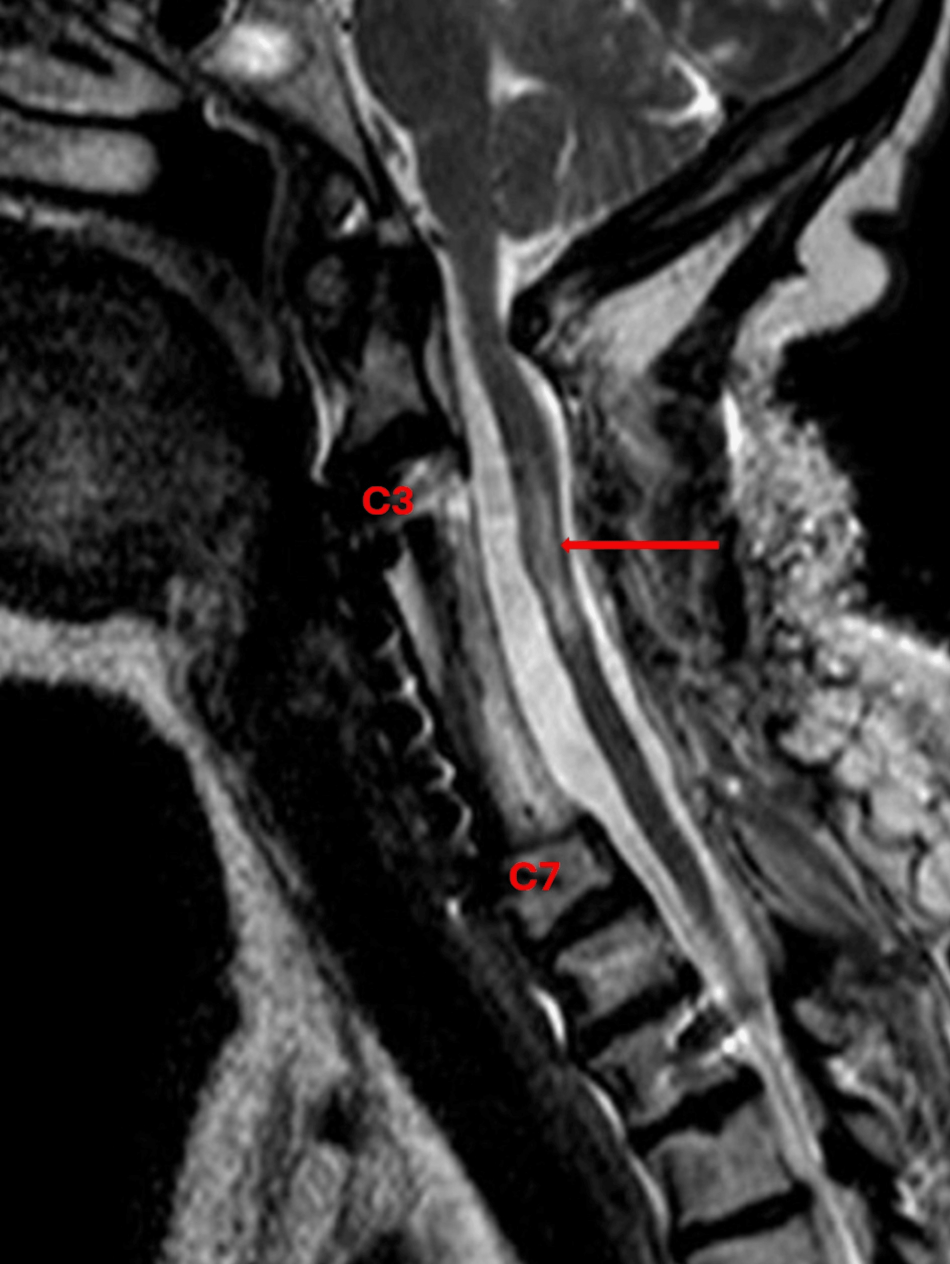
Postoperative cervical spine sagittal T2-weighted MRI. Red arrow shows a hyperintense signal at C4-C5 and surrounding spinal edema.

The patient was safely extubated, with adequate tidal volume, intact airway reflexes, and spontaneous ventilation. She was then transported to the post-anesthesia care unit (PACU). Once the patient was awake and alert, the on-call anesthesia team performed an initial evaluation, which revealed a motor strength of 0/5 in all four extremities. Upon discharge from PACU, her motor strength improved to 1/5 in both upper and lower extremities. She was transferred to the neuro-intensive care unit (Neuro-ICU) for recovery and further management. On postoperative day (POD) one, a cervical spine sagittal T2-weighted MRI was performed, which displayed a new hyperintense signal along C4-C5 (Figure 4). The patient remained in the Neuro-ICU, where she was closely monitored for blood pressure (BP) and glucose control. During her stay, the patient’s motor strength improved to 2/5 bilaterally in the upper extremities, although lower extremities remained the same with a 1/5 motor strength. The patient was discharged on POD 12 to an inpatient rehabilitation facility.

Following inpatient rehabilitation, a neurological examination at one-month follow-up showed improvement of both upper and lower extremity strength to 3/5 bilaterally, with 2+ reflexes throughout. Her condition remained unchanged and stable at her three-month follow-up. A timeline of neurological changes is included in Table 2.

**Table 2:** Timeline of motor and sensory findings throughout the perioperative period. Motor findings are summarized using the Medical Research Council (MRC) Escale. Sensory findings are summarized using the American Spinal Injury Association (ASIA) Neurologic Assessments. UE: upper extremities; LE: lower extremities

Timeline	Preoperative	PACU (POD 0)	Neuro ICU (POD 1)	Discharge (POD 12)	Rehab Follow-Up (1 & 3 months)
Motor Function (MRC)	UE and LE: 4/5 bilaterally	UE and LE: 0/5 → 1/5 bilaterally	UE: 2/5 bilaterally	UE: 3/5 bilaterally	UE and LE: 3/5 bilaterally
			LE: 1/5 bilaterally	LE: 2/5 bilaterally	
Sensory Function (ASIA)	Normal	Normal	Normal	Normal	Normal

## Discussion

We present the case of a female patient with type 2 diabetes and perioperative hyperglycemia who developed WCS following a C4-C5 corpectomy for symptomatic multilevel cervical stenosis. Our patient exhibited the hallmark radiologic feature of a T2W-MRI hyperintense signal in the context of unexplained neurological deficits after cervical spinal cord decompression.

The use of multimodal intraoperative neurophysiological monitoring (IONM), including SSEPs and MEPs, is the common practice for specific detection of neurologic injury during surgical procedures [11]. Early detection of changes in these monitoring modalities allows the operative team to optimize patient hemodynamics and initiate steroid therapy. In our case, close surveillance enabled the detection of abrupt loss of both MEPs and SSEPs, raising concern for ischemia-reperfusion injury. Because these findings were detected within a three-hour period, and no mechanical causes were identified, the NASCIS II protocol was initiated with the administration of methylprednisolone [12]. The neuroprotective effects of high-dose steroid therapies are attributed to the inhibition of lipid peroxidation and inflammatory cytokines, two key processes implicated in neuronal and microvascular membrane injury [13]. However, in a meta-analysis evaluating the use of high-dose methylprednisolone following acute traumatic SCI, Liu et al. [14] found no evidence of an association with improved neurological recovery. Moreover, these authors did not recommend this practice because it increased the risk of adverse events, including respiratory tract infection and sepsis, among others. Although the practice remains controversial, the use of high-dose methylprednisolone continues to be one of the few structured, widely adopted protocols for the management of acute SCI.

An essential factor in understanding the pathophysiology of SCI is the integrity of the blood-spinal cord barrier (BSCB), a specialized extension of the blood-brain barrier composed, in part, of non-fenestrated capillary endothelial cells [15]. The BSCB maintains spinal cord homeostasis by tightly regulating the exchange of solutes between the systemic circulation and neural tissue [16]. Because spinal cord perfusion depends on the balance between MAP and cerebrospinal fluid pressure, maintaining adequate perfusion is critical to ensure optimal oxygen and nutrient delivery. Disruption of this balance can compromise BSCB function, resulting in spinal cord edema, leukocyte infiltration, and increased levels of inflammatory and oxidative stress responses [16]. Together, these pathological processes may contribute to ischemia-reperfusion injury and neurological deterioration after surgical decompression [15]. In WCS, where ischemia-reperfusion injury is thought to play a central role, excessive increases in perfusion pressure should be avoided, as they may intensify oxidative damage through enhanced production of oxygen-derived free radicals during reperfusion and may also exacerbate spinal cord swelling [4]. By contrast, low perfusion pressures during early reperfusion exert neuroprotective effects in the spinal cord of rabbits undergoing thoracic aortic surgery [17]. These findings suggest that the magnitude of reperfusion, rather than the process itself, may determine neurological outcomes, underscoring the importance of precise intraoperative blood pressure management to prevent both inadequate and excessive perfusion. Although animal data suggest that low reperfusion pressures may be beneficial, in our case, MAP was maintained at approximately 90 mmHg to ensure adequate spinal cord perfusion in accordance with current traumatic SCI guidelines. Moreover, in WCS, optimal MAP targets remain uncertain.

In a recent study of patients with acute SCI, it was observed that those treated with high-dose methylprednisolone were more likely to develop hyperglycemia compared to patients who did not receive corticosteroids [18]. Hyperglycemia promotes excessive production of ROS, free fatty acids, and pro-inflammatory mediators, which lead to endothelial cell damage and contribute to the destruction of the BSCB [19]. Despite intraoperative insulin administration, glycemic control in our patient remained suboptimal, potentially increasing oxidative stress and further contributing to vascular deterioration. Consequently, diabetic patients who require intraoperative corticosteroids may be at a heightened risk for ischemia-reperfusion-related complications during spinal surgery, including the development of WCS. Moreover, the complex pathophysiology of diabetes creates unique perioperative challenges in SCI management, as hyperglycemia has been associated with poorer neurological recovery [20]. Therefore, in our patient, the combination of poorly controlled diabetes and high-dose corticosteroid administration may have contributed to the pathophysiological development of WCS and subsequent neurological presentation. This emphasizes the importance of thorough preoperative risk assessment and individualized strategies to address modifiable risks in diabetic patients undergoing spinal cord decompression surgery.

## Conclusions

Given the rarity of WCS, the uncertainty surrounding its risk factors, and the lack of standardized treatment algorithms, preventing this complication during spinal cord decompression remains challenging. Nevertheless, several perioperative strategies may help mitigate risks, including meticulous surgical techniques, vigilant intraoperative neuromonitoring, careful MAP optimization, and, potentially, optimal glycemic control. While poorly controlled diabetes and steroid use may plausibly contribute to WCS, current data support these factors only as hypothetical contributors. Future research should focus on identifying clearer predisposing factors and developing evidence-based protocols to guide the prevention and management of this devastating condition.
